# Adipose-derived mesenchymal stem cells with hypoxic preconditioning improve tenogenic differentiation

**DOI:** 10.1186/s13018-021-02908-2

**Published:** 2022-01-28

**Authors:** Xing Guo, Denghua Huang, Dan Li, Longfei Zou, Hui Lv, Yuanhui Wang, Meiyun Tan

**Affiliations:** 1 Department of Burns and Plastic Surgery, The Affiliated Hospital of Southwest Medical University, Luzhou, China; 2 Department of Orthopedic Surgery, The Affiliated Hospital of Southwest Medical University, Luzhou, China

**Keywords:** Adipose-derived mesenchymal stem cells, Tendon, Tissue engineering

## Abstract

**Background:**

Adipose-derived mesenchymal stem cells (ADSCs), as seed cells for tendon tissue engineering, are promising for tendon repair and regeneration. But for ADSCs, diverse oxygen tensions have different stimulatory effects. To explore this issue, we investigated the tenogenic differentiation capability of ADSCs under hypoxia condition (5% O_2_) and the possible signaling pathways correspondingly. The effects of different oxygen tensions on proliferation, migration, and tenogenic differentiation potential of ADSCs were investigated.

**Methods:**

P4 ADSCs were divided into a hypoxic group and a normoxic group. The hypoxic group was incubated under a reduced O_2_ pressure (5% O_2_, 5% CO_2_, balanced N_2_). The normoxic group was cultured in 21% O_2_. Two groups were compared: HIF-1α inhibitor (2-MeOE2) in normoxic culturing conditions and hypoxic culturing conditions. Hypoxia-inducible factor-1α (HIF-1α) and VEGF were measured using RT-qPCR. Specific HIF-1α inhibitor 2-methoxyestradiol (2-MeOE2) was applied to investigate whether HIF-1α involved in ADSCs tenogenesis under hypoxia.

**Results:**

Hypoxia significantly reduced proliferation and migration of ADSCs. Continuous treatment of ADSCs at 5% O_2_ resulted in a remarkable decrease in HIF-1α expression in comparison with 20% O_2_. Additionally, ADSCs of hypoxia preconditioning exhibited higher mRNA expression levels of the related key tenogenic makers and VEGF than normoxia via RT-qPCR measurement (*p* ˂ 0.05). Furthermore, the effects of hypoxia on tenogenic differentiation of ADSCs were inhibited by 2-MeOE2. Hypoxia can also stimulate VEGF production in ADSCs.

**Conclusions:**

Our findings demonstrate that hypoxia preconditioning attenuates the proliferation and migration ability of ADSCs, but has positive impact on tenogenic differentiation through HIF-1α signaling pathway.

## Introduction

Tendons are responsible for the transmission of forces to the skeleton to generate locomotion. As a result, they frequently experience biomechanical injuries. The inherent capability of a tendon to heal is, however, unsatisfactory because of its hypocellular and hypovascular nature. Additionally, the formation of scar tissue in a tendon reduces its mechanical properties, making it susceptible to reinjures [1]. Several methods have been reported for the repair of injured tendons but their curative effects remain limited, because of the low mechanical strength and high incompleteness of tendon [2–5]. Therefore, new therapeutic strategies still need to be developed for the treatment of injured tendons.

Mesenchymal stem cells (MSCs) can differentiate into several mesenchymal tissues (e.g., bone, adipose, tendon) and may be used for the regeneration and repair of these tissues [6–9]. Bone marrow mesenchymal stem cells (BMSCs) are frequently used for such purposes, but these cells are highly scarce. Furthermore, BMSCs may undergo severe apoptosis because of their poor tolerance of the hypoxic conditions in the injured, and thus ischemic, site [10].

Adipose-derived mesenchymal stem cells (ADSCs) were first discovered in the 1980s. ADMSCs appear to be ideal seed cells for tissue regeneration owing to their relative abundance, convenient access to material, low immunogenicity, self-renewal, and multi-lineage differentiation potential. Transplantations of BMSCs or ADSCs have been reported to achieve encouraging effects in pathological and injured tendons [11–14]. Behfar et al. [15] reported that ADSCs were more suitable for tendon repair than BMSCs.

Currently, in vitro amplification of MSCs still faces many technical challenges [16]. One study found the oxygen concentration range of adipose tissue was 2–8% [17]. For ADSCs, hypoxia can enhance their survival and is non-oncogenic [18–20]. In fact, hypoxia was reported to play important roles in the proliferation, migration, differentiation, and growth factor secretion of ADSCs [21–25]. Therefore, it is still necessary to develop new treatment strategies to treat tendon injury. Several studies found that, hypoxia precondition increased the therapeutic potential of MSCs via induction of cellular prion expression [26, 27]. As opposed to vascular-rich tissues, the hypovascular nature of tendons results in low local oxygen levels [28]. A hypoxic microenvironment has been suggested to be essential for the healing of a tendon [29]. Recently, Yu et al. [30] reported that hypoxia (2% O_2_) improves the tenocyte differentiation of ADSCs. However, the tenogenic impact of hypoxia on ADSCs, notably 5% O_2_, remains largely unexplored. Hypoxia affects cellular functions by modulating transcription factor-α (HIF-1α), which binds to HIF-1 response elements and regulates mitochondrial metabolism and endoplasmic reticulum stress [31]. However, the cellular and molecular mechanisms underlying the effects of hypoxia on ADSCs are not fully understood.

A tendon injury represents a relatively severe hypoxic and ischemic microenvironment. It is well known that, VEGF plays an important part in tissue repair processes. Under impaired blood circulation, VEGF activates tissue neovascularization to restore the oxygen supply to local tissues. Several studies found that, culture of ADSCs under low oxygen tensions (1% or 2% O_2_) significantly enhanced their VEGF expression to promote angiogenesis [32–34]. However, long-term treatment of ADSCs under 5% O_2_ tension has rarely been reported.

In this study, we aimed to investigate whether the low oxygen O_5_ tension could enhance the proliferation, migration, and tenogenic differentiation potential of ADSCs in vitro. We further studied, by inhibiting HIF-1α inhibitor, the role of HIF-1α signaling pathway in the ton effect of tenogenic differentiation of ADSCs under low O_5_ tension by using HIF-1α inhibitor. We suggest that, findings of the present study may provide useful information for the development of more effective showed would be benefit to the tendon tissue regeneration techniques based on ADSCs engineering.

## Materials and methods

### Isolation and culture of rat adipose-derived mesenchymal stem cells

All animal experiments were approved by the Animal Research Ethics Committee, Southwest Medical University. Male Sprague–Dawley rats (3–4 weeks, 160–250 g) were purchased from Laboratory Animal Center of Southwest Medical University, ADSCs were isolated from the inguinal area as described previously [1]. Briefly, adipose samples aseptically collected from the area were mechanically minced into approximately 1 mm^3^ pieces and incubated in 0.1% type-I collagenase (Sigma-Aldrich, St. Louis, MO, USA) at 37 °C f or 1 h. The resulting single-cell suspension was centrifuged at 300 g for 10 min; the supernatant was discarded; and the sediment was suspended in DMEM/F12 medium (Hyclone, Logan, UT, USA) supplemented with 10% fetal bovine serum (FBS), and 1% penicillin–streptomycin. The ADSCs thus isolated were incubated (37 °C, 5% CO_2_) for how long to form colonies with a medium changed every 3 d. The cells were sub-cultured when they reached 80–90% confluence. Cells at passage 3–5 (P3–P5) were used for subsequent experiments.

### Characterization of ADSCs

Surface markers expressed by ADSCs were analyzed by flow cytometry (FC). ADSCs were suspended in PBS, then incubated in DMEM containing antibodies to anti-CD29, anti-CD31, anti-CD45, or anti-CD90 (BD, USA), respectively, for 30 min at room temperature. Then, the cells were analyzed by FC. Multi-lineage differentiation potential of ADSCs was evaluated separately by culture in adipogenic, osteogenic, or chondrogenic differentiation media (Cyagen Biosciences, Santa Clara, CA, USA). Briefly, P3 ADSCs (2 × 10^4^ cells*/*well) were incubated in 6-well plates. For adipogenetic differentiation, after the ADSCs reached 100% confluence, the medium was replaced with rat ADSCs adipogenic differentiation medium. After 21 d, adipogenic differentiation was evaluated by Oil Red O staining. For osteogenic differentiation, after the cells reached 60–70% confluence, the medium was replaced with the osteogenetic differentiation medium. Then, the cells were cultured in 21% O_2_. After 21 d, the osteogenic differentiation was evaluated by Alizarin Red staining. For chondrogenic differentiation, a cartilage sphere culture was performed. After 28 d, the cartilage sphere was fixed in 4% formalin, embedded in paraffin, and Toluidine Blue staining was performed.

### Experimental design

P4 ADSCs were divided into a hypoxic group and a normoxic group. The hypoxic group was incubated under a reduced O_2_ pressure (5% O_2_, 5% CO_2_, balanced N_2_) in a tenogenic medium [DMEM, 15% FBS, 100 mM ascorbic acid, 100 nM dexamethasone, 100U/mL penicillin, 100 µg/mL streptomycin, 2 mM sodium pyruvate (Gibco), and 2 mM L-glutamine (Gibco)] [2]. The normoxic group was cultured in 21% O_2_ in the same medium. In Addition, two groups were compared: HIF-1α inhibitor (2-MeOE2) in normoxic culturing conditions and hypoxic culturing conditions.

### Cell proliferation assay

P3 ADCSs were suspended in a tenogenic medium, seeded into a 96-well plate, and incubated separately under normoxic (21% O_2_) or hypoxic (5%O_2_) conditions. On days 1, 3, 5, and 7, cell numbers were assayed with cell-counting kits (CCK‐8, Dojindo Laboratories, Kumamoto, Japan). More specifically, 2 h before assay, the medium was replaced with 100 µL of DMEM/F12 and 10 µL of CCK-8. After incubation for 2 h, the optical density at 450 nm was measured.

### Scratch assay

For scratch assay, ADSCs were seeded into 6-well plates (5 × 10^5^ cells/well) and incubated in induction medium for 24 h. A 20-µL pipette tip was used to create a scratch in a confluent cell monolayer. ADSCs were rinsed twice with PBS to remove the floating cells; 2 ml of serum-free medium was added to the well; and the cells were further cultured under the aforementioned normoxic or hypoxic condition. At 0 and 24 h, the scratched region was imaged with an inverted light microscope. For each group, the migration rate of ADSCs was measured by image analysis of 10 micrographs recorded from 5 independent samples.

### Pharmacological HIF-1α inhibition study

To inhibit the expression of HIF-1α, the specific inhibitor of HIF-1α (2-MeOE2), which was purchased from Selleck Chemicals LLC, was added into the induced culture medium for 24 h in hypoxic conditions, as described previously [35]. A concentration gradient test was performed to determine the minimum effective concentration (MEC) of the inhibitor. The 2-methoxyestradiol was used at concentration of 0, 5, 10, and 15 µM, respectively. ADSCs were then treated with 2-methoxyestradiol at MEC in both normoxia and hypoxia samples.

### In vitro immunofluorescence staining

The treated ADSCs were seeded on glass slides pretreated with polylysine, and the slides were transferred into 6-well plates. After culture for 7 d, the cells were fixed in 4% paraformaldehyde, permeabilized with 0.1% Triton X-100, and blocked using 5% goat serum. The cells were probed with primary antibody to rabbit tenomodulin (1:50; Abcam; 4 °C, overnight), and then horseradish peroxidase-labeled goat IgG to rabbit IgG (FITC) (1:500; Abcam; 37 °C, 1 h). Finally, they were counterstained with DAPI at room temperature for 10 min.

### RNA isolation, reverse transcription, and RT-qPCR analysis

To assess the differentiation of ADSCs on tendon-lineage differentiation, quantitative PCR analyses were performed on ADSCs cultured under 5% or 21% O_2_ for 0, 3, and 7 days. Total cellular RNA was extracted with RNeasy Mini kits (Qiagen, Valencia, CA, USA). The mRNA was reverse-transcribed to cDNA with ReverTra AcqPCR RT Master Mix kits, and the target cDNA was amplified with SYBR Green Realtime PCR Master (both Toyobo, Osaka, Japan) that was used to amplify the target cDNA. Target gene expression was analyzed by Mix Quantitative PCR analysis using s was then performed to detect the target gene expression. The primers (Sango Biotech, Shanghai, China) targeting TNMD, DCN, SCX, VEGF, HIF-1α, and the control glyceraldehyde-3-phosphate dehydrogenase (GAPDH, internal reference) were designed by Sango Biotech (Shanghai, China).

The PCR primers used were sequences are as follows: SCX (5’′-AGATCGCAAGCTCTCCAAGA -3′’ and 5′’- CAGGCTTCACCCACCAGTAG -3′’), TNMD (5′’- TCTGGAGATTTGCGACAATG -3′’ and 5′’- TCGCTGGTAGGAAAGTGAAGA -3′’), DCN (5′’- CCTTGCAGGGAATGAAGGGT-3′’ and 5′’-TGTTGCCATCCAGATGCAGT-3′’), HIF-1α(5′’-GAACCCATTCCTCATCCATCAAAC -3′’ and 5′’- TCTTCTGGCTCATAACCCATCAAC -3′’), VEGF (5′’- AATTGAGACCCTGGTGGACA -3′’ and 5′’-ACTCCAGGGCTTCATCATTG-3′’), GAPDH (5′’-CCCCCAATGTATCCGTTGTG-3′’and5′’-TAGCCCAGGAT GCCCTTTAGT -3′’). The cycling PCR programs comprised conditions were: performed: 95 °C for 30 s, 40 cycles at 95 °C for 5 s, optimal annealing temperature for 10 s, and then 72 °C for 15 s. The relative expression levels of target genes were determined using the 2^−△△Ct^ formula and normalized to GAPDH.

### Statistical analysis

All data were reported as mean ± standard deviation (SD) from independent experiments. Statistical comparisons of the treatment and control groups. Data from the two groups were compared by using Student’s* t*-test. Data recorded under, > 2 experimental conditions were analyzed by one-way analysis of variance (ANOVA) and Dunnett’s post hoc test. A *p* ˂ 0.05 was considered statistically significant, and *p* ˂ 0.01 highly significant.

## Results

### Characterization of ADSCs

**Fig. 1:**
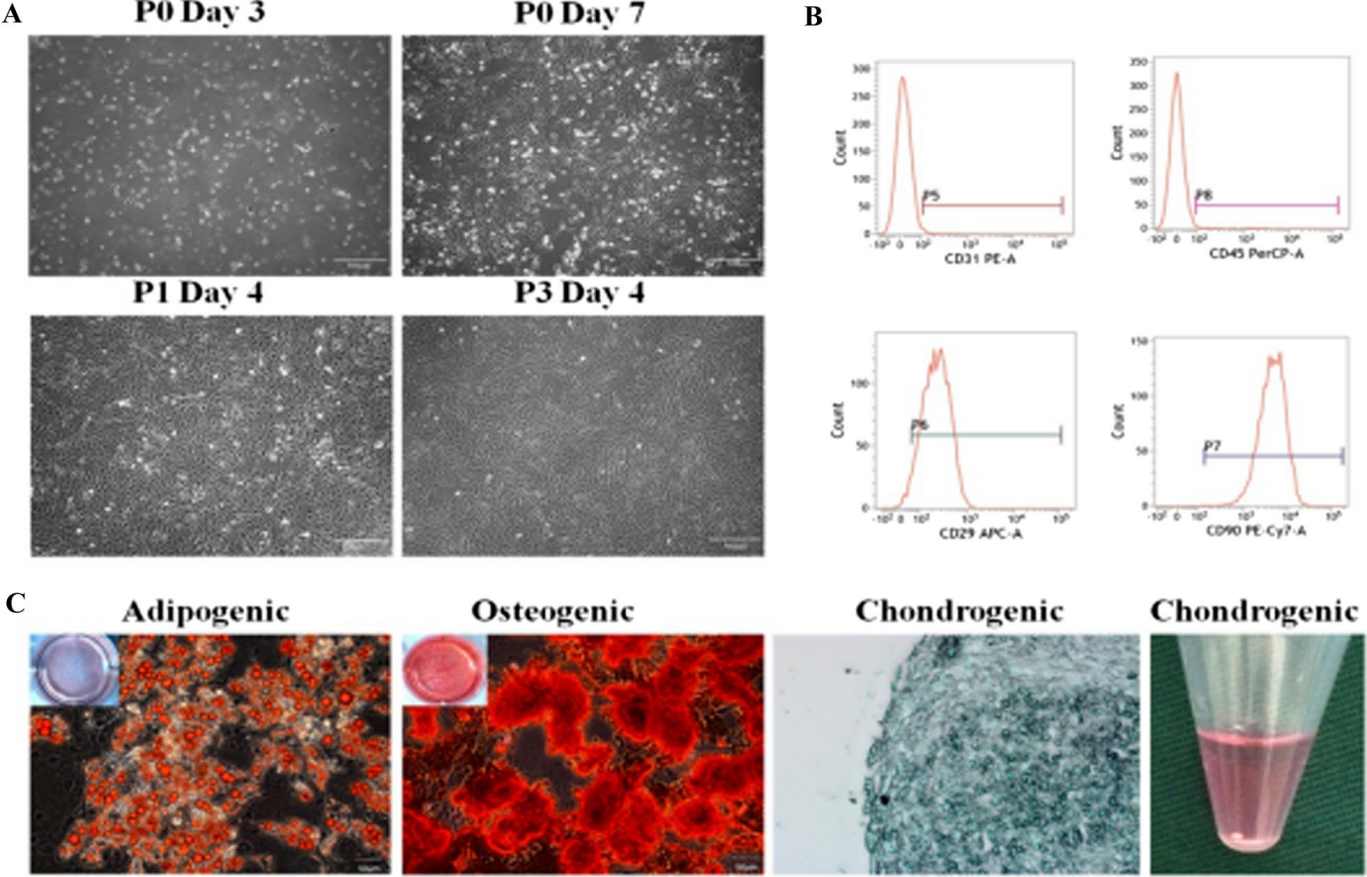
Morphological and characteristic observation of ADSCs. **A** The ADSCs exhibited characteristic fibroblast-like morphology. Magnification 4 × , *bar* = 500 μm. **B** Cell surface marker expression of ADSCs was determined by flow cytometric analysis. Cultured ADSCs were positive for CD29 and CD90 but negative for CD31 and CD45. **C** Multi-lineage differentiation assays were used to detect the differentiative potential of ADSCs. The cultured ADSCs were under adipogenic, osteogenic, and chondrogenic induction and stained with Oil Red O, Alizarin Red, and Alcian Blue, respectively. Magnification 200 × , *bar* = 100 μm

Flow cytometry (FC) found that, the ADMSCs were negative for CD31 (1.37% ± 0.32%) and CD45 (1.64% ± 0.24%), and positive for CD29 (96.79% ± 1.01%) and CD90 (99.93% ± 1.05%) (Fig. 1B).

Multi-differentiation assays showed that, after culture in the presence of specific inductors, the cells successfully differentiated into adipogenic, osteogenic, and chondrogenic lineages (Fig. 1C). After osteogenic induction, Alizarin red S staining detected the formation of mineralized nodules. After adipogenic induction, lipid droplets in the cells were intensely positively stained by intense Oil Red O. After chondrogenic induction, Alcian blue staining indicated the presence of internal acidic polysaccharides in the induced cartilage ball.

### Cell proliferation

**Fig. 2:**
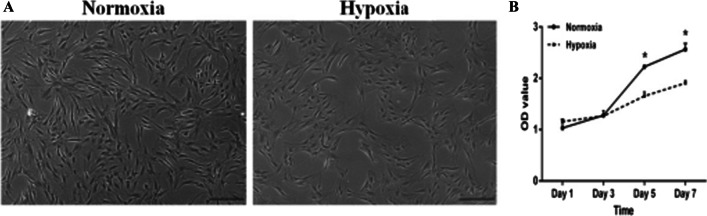
Hypoxia suppressed the proliferation of ADSCs. **A** ADSCs kept 7 days under normoxic or hypoxic (5% O_2_) conditions. **B** Proliferation rates of ADSCs further confirmed using CCK-8 assay indicated significantly reduced after 5 and 7 days under hypoxic condition. *p ˂ 0.05. Magnification 10 × , *bar* = 200 μm, OD, optical density

We evaluated the proliferation of ADSCs under normoxic and hypoxic conditions between days 1 and 7. Light microscopy showed that, after culture for 7 d, the hypoxic group had a lower cell density than did the normoxia group (Fig. 2A). Quantitative assay with CCK-8 kits found that, on day 1 after induction, the two groups had similar cell numbers (*p* > 0.05). Between days 3 and 5, both groups increased substantially in cell number; on day 5, the normoxia group is higher than the hypoxia group, and the difference was highly significant (*p* ˂ 0.05). In addition, we found that there was the maximum proliferation rate on day 5. Interestingly, compared with on day 7, the proliferation ability of ADMSCs of both groups distinctly reduced and no significant differences were presented between the two groups (*p* > 0.05) (Fig. 2B).

### Hypoxia suppresses ADSCs migration

**Fig. 3:**
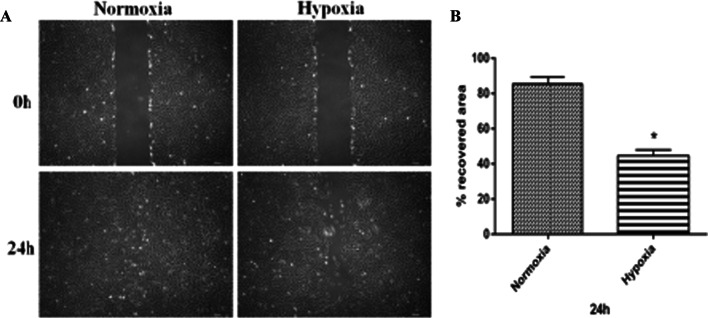
Hypoxia inhibited the migration of ADSCs. **A** Monolayers of ADSCs were wounded with pipette tips and incubated for 24 h at 37 °C. **B** The average percentages of recovered area obtained from normoxia and hypoxia groups. *p ˂ 0.05, Magnification 10 × , *bar* = 200 μm

Scratch experiments were conducted to evaluate the effect of hypoxia on the activation of ADSCs migration. After 24 h, the normoxia group exhibited a remarkably higher ADSC migration compared with the hypoxia group (Fig. 3A). Image analysis indicated that, the scratch area of the normoxia group was covered by ADSCs after 24 h, compared with the hypoxia group (Fig. 3B). The difference was highly significant (*p* ˂ 0.05).

### Hypoxia promotes the tenogenic differentiation potential of ADSCs

**Fig. 4:**
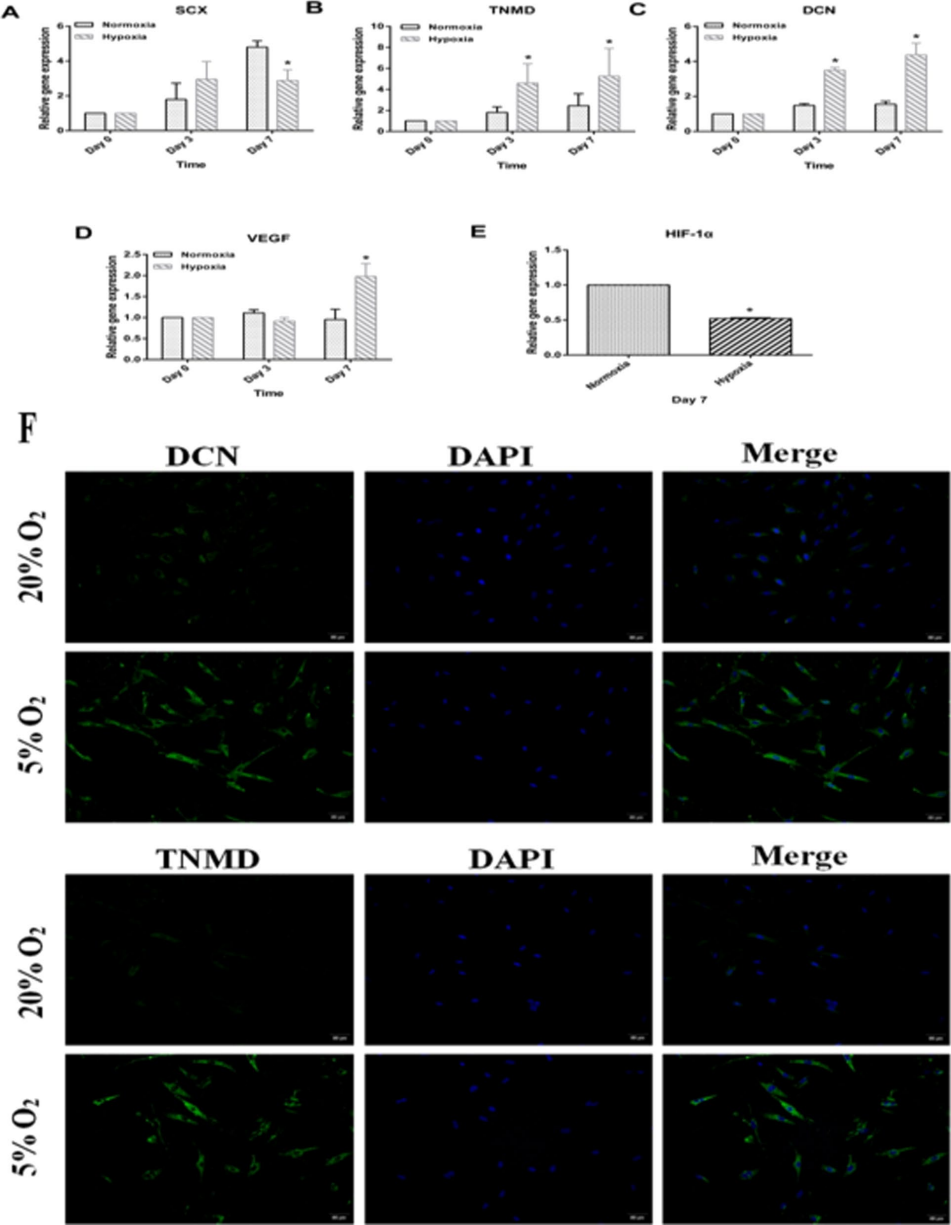
The effects of hypoxia on HIF-1α expression and the tenogenic differentiation of ADSCs. The relative genes expression of **A** SCX, **B** TNMD, **C** DCN, **D** VEGF, and **E** HIF-1α were detected by real-time PCR analysis at different processing time points. All results were presented as mean ± SD. *p ˂ 0.05, compared with control. (**F**) Representative micrographs showed with immunofluorescent staining for DCN (green) and TNMD (green), and DAPI- counterstained nuclei (blue) in normoxic and hypoxic groups

We investigated the tenogenic differentiation potential of the ADSCs by incubation in the tenogenic differentiation medium (TDM) under hypoxic or normoxic conditions for up to 7 d followed by RT-qPCR. It was found that, hypoxic preconditioning significantly upregulated the expression levels of tenocytes-associated marker genes, including SCX (i.e., a key transcription factor) [36], TNMD (i.e., a surface marker [37], and DCN (i.e., an extracellular matrix protein) [38] at all time points (*p* < 0.05) (Fig. 4A–C). Additionally, on day 7, the hypoxia group emitted a markedly stronger fluorescence indicating TNMD than did the normoxia group (Fig. 4F), consistent with the RT-qPCR results. Collectively, these findings confirmed that hypoxia preconditioning enhanced the tenogenic differentiation of the ADSCs.

### Hypoxia downregulates HIF-1α levels in ADSCs

To investigate the effect of hypoxia on HIF-1α expression in the ADSCs, we measured the gene expression level of HIF-1α by RT-qPCR on day 7. It was found that, compared with the normoxia group, the HIF-1α expression in the hypoxia group was significantly reduced by 50% (*p* < 0.05) (Fig. 4E).

### Hypoxia-induces tenogenic differentiation of ADSCs via inducing HIF-1α expression

**Fig. 5:**
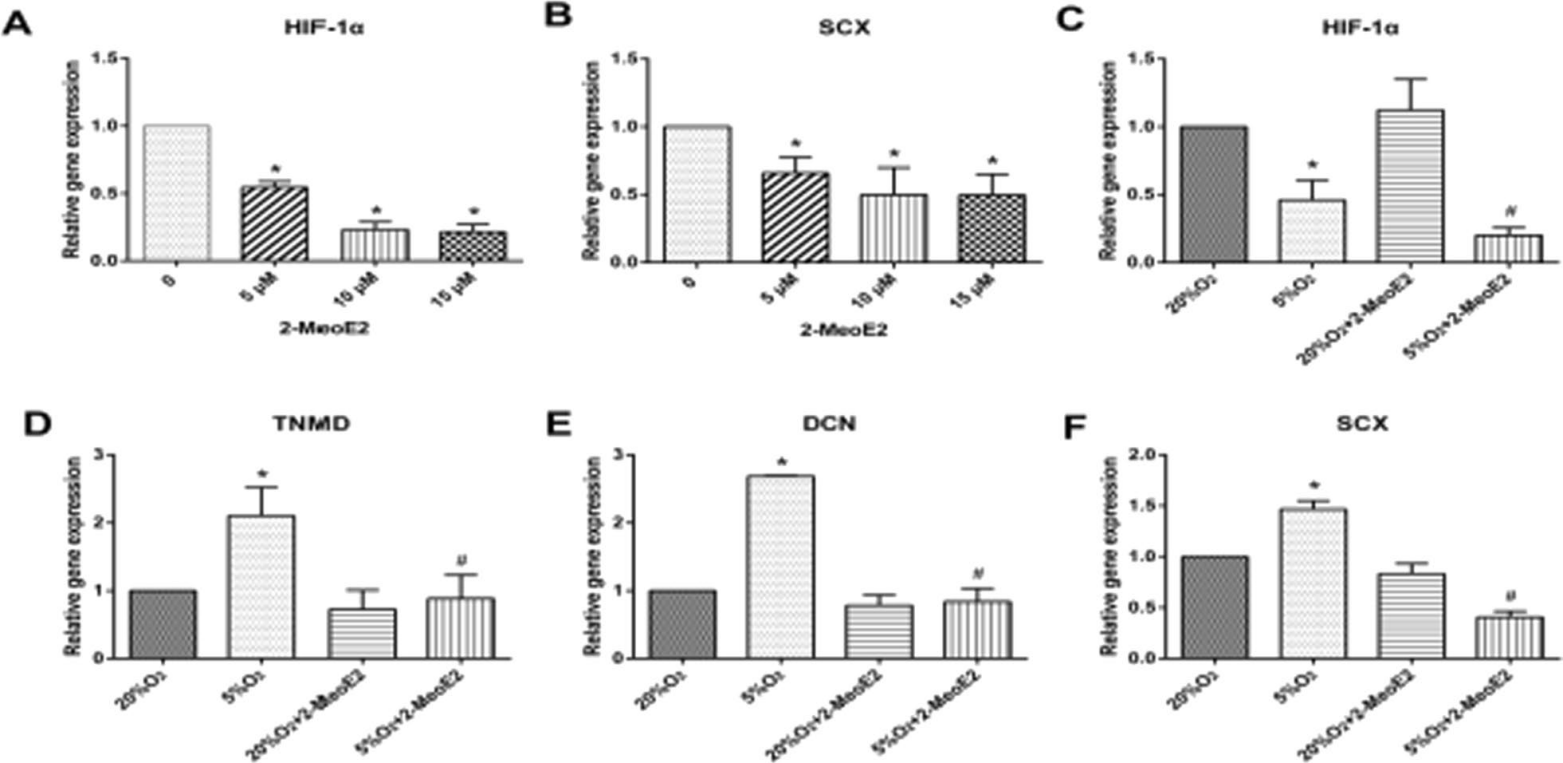
Effects of HIF-1α inhibitor on HIF-1α activity and the tenogenic differentiation of ADSCs. **A**, **B** The ADSCs supplemented with 2-MoeE2 at indicated concentrations. The HIF-1α and SCX expression were detected with real-time PCR analysis by concentration gradient test.*p ˂ 0.05, compared with control. **C**–**F** The ADSCs were treated with 10 μM of 2-MoeE2 under normoxia or hypoxia for 24 h, the expression levels of (**C**) HIF-1α, (**D**) TNMD, (**E**) DCN, and (**F**) SCX were assessed by RT-PCR. All results were presented as mean ± SD. *p ˂ 0.05, compared with the 20% O_2_ culture group without 2-MoeE2 treatment; #*p* ˂ 0.05, compared with the 5%O_2_ culture group without 2-MoeE2 treatment

To investigate whether HIF-1α is involved in the tenogenic differentiation of ADMSCs, the cells were treated with 2-MeOE2 (i.e., HIF-1α inhibitor) for 24 h. CT-qPCR found that, the 2-MeOE2 treatment regulated HIF-1α and SCX expression in a concentration-dependent manner (5–15 µM) (Fig. 5A, B). After treatment with 5, 10, or 15 µM2-MeOE2, the HIF-1α expression decreased to approximately 1/3, 1/5, and 1/5 of the untreated control, respectively. This suggests that, 10 µM 2-MeOE2 was more effective in inhibiting the HIF-1α activity than did 5 µM, but further elevation to 15 µM did not produce a further increase in HIF-1α inhibition. Therefore, 10 µM 2-MeOE2 was used in subsequent experiments.

Then, 10 µM 2-MeOE2 was added to the ADMSC cultured medium, and incubated under normoxic or hypoxic conditions for 24 h followed by RT-qPCR. It was found that, when 2-MeOE2 was absent, the expression levels of HIF-1α, TNMD, DCN, and SCX (i.e., tendon-associated genes) in the hypoxia group were significantly enhanced compared with the normoxia group (Fig. 5C–F) (all *p* < 0.05). In comparison, when 10 µM 2-MeOE2 was added to the medium, the differences in the expression levels of TNMD and DCN between the two groups became statistically insignificant (both *p* > 0.05). Moreover, the expression level of SCX in the hypoxia group became significantly lower than the normoxia group. Taken together, these findings indicate that, the hypoxic conditions stimulated the tenogenic differentiation of the ADMSCs via inducing HIF-1α expression.

### Hypoxia promotes VEGF production in ADSCs

We further examined the effect of hypoxic conditions on the VEGF expression of the ADSCs. RT-qPCR found no statistically significant difference in VEGF between the two groups on days 0 and 7 (both *p* > 0.05) (Fig. 4D). On day 7, however, the VEGF expression level in the hypoxia group was significantly higher than that in the normoxia group (*p* = 0.0*,). However, hypoxia slightly decreased VEGF release at 3 days, which has no significant difference (*P* > 0.05).

## Discussion

ADSCs are widely used in tendon tissue engineering. Generally, the cells are cultured in vitro under normoxic conditions (21% O_2_). These conditions, however, deviate from the microenvironment experienced by tenocytes, which inherently lacks vascularization and thus oxygen supply. An injured tendon is an even more ischemic and hypoxic microenvironment. Despite this, few studies have investigated the application of hypoxic conditions to in vitro ADSCs culture for tendon tissue engineering. For such purpose, it is important to sufficiently understand the characteristics of ADSCs in hypoxic conditions.

The present study found that, hypoxia (5% O_2_) reduced the proliferation and migration of the ADSCs (Figs. 2, 3). In comparison, 5% O_2_ promoted the tenogenic differentiation of the ADSCs via an HIF-1α signaling pathway, as demonstrated by inhibiting experiments (Fig. 5). VEGF is well known to play a pivotal role in angiogenesis. VEGF secretion by ADSCs is highly limited under normoxic culture conditions. Interestingly, we found that hypoxia stimulated the VEGF gene expression in ADSCs (Fig. 4). To our best knowledge, this is the first report exhibiting that augment of ADSCs tenogenic differentiation potential by 5% oxygen concentration pretreatment might be directly or indirectly owing to the HIF-1α signaling pathway.

The response of cells to the decrease in ambient O_2_ is an elementary adaptation reaction, which is critical for cell survival. Many studies reported that the proliferation and migration of cells in hypoxic microenvironments can vary substantially, depending on the different cell lines and O_2_ level [39]. Our results showed that ADSCs had significantly lower proliferation and migration under 5% O_2_ (Fig. 2). It is noted that, conflicting results have been reported [40–43] regarding whether 5% O_2_ enhances or reduces ADSC proliferation and migration, although the majority of studies observed reduced proliferation and migration. The differences from those studies may be related to different experimental conditions (e.g., culture conditions, exposure time, grow factors). Nevertheless, the underlying factors remain poorly understood.

Yu et al. [30] reported that it has confirmed that reduced oxygen tension (2% O_2_) provides an enhancement for the tenogenic potential of ADSCs. It is known that the tenogenic induction increases the upregulation of TNMD, SCX, and DCN during tenogenesis. So, coincident with previous research, in the present study, we also demonstrated the important role of the hypoxia preconditioning on the tenogenic differentiation of ADSCs (Fig. 5). The data showed that, in both hypoxia and normoxia treatment groups, the expression genes levels of all analyzed tested tenogenic marker genes exhibited an increased tendency, which is dependently related to time (except SCX) (Fig. 5). Moreover, hypoxia-treated group was significantly higher than normoxia treatment. Compared to normoxia group, hypoxia made a lower mRNA expression level of SCX at 7 days. We deduced that, for SCX, exposure to 5% O_2_ for 7 d was so long that its expression was inhibited by hypoxia, but the underlying mechanisms remain to be determined.

HIF-1α, a sensitive molecule in the cell reaction regulation mechanism, facilitates the adaptation of MSCs to hypoxic conditions. HIF-1α was reported to participate in the suppression and enhancement of osteogenic, adipogenic, and tenogenic differentiation of ADSCs [30, 35, 44, 45]. In our study, HIF-1α was significantly downregulated by the hypoxic condition on days 1 and 7 (Fig. 2). The expression levels of tenogenic differentiation-related genes (i.e., TNMD, SCX, DCN) were remarkably upregulated in the hypoxia group on days 3 and 7 (Fig. 4). Our results showed that 5% O_2_ reduced HIF-1α gene expression and increased the tenogenic differentiation of the ADSCs, indicating that HIF-1α was closely related to the regulation of tenogenic differentiation of ADSCs under hypoxia. Hypoxia-mediated upregulation of these genes was inhibited by HIF-1α inhibitor 2-MeOE2 probably via a process. For example, 2-MeOE2 may trigger microtubule depolymerization, and subsequently inhibit HIF-1α nuclear accumulation and transcriptional activity.

The majority of earlier studies reported that, under hypoxic conditions, HIF-1α was activated and exhibited a high expression level, although a few studies observed opposite trends [30, 46]. Pogodina et al. found that, under 5% O_2_, HIF-1α expression in ADSCs increased initially and subsequently decreased within 24 h [46]. Our findings indicated that continuous exposure of ADSCs to 5% O_2_ resulted in a remarkable decrease in HIF-1α expression (vs. 20% O_2_) on days 1 and 7, consistent with the former trend. Nevertheless, the tenogenic markers (i.e., TNMD, SCX, DCN) were significantly increased at the same time and oxygen concentration. We suggest that, during long-term exposure to hypoxia, the expression of HIF-1α after activation of the ADSCs tenogenic marker genes is likely regulated by negative feedback regulation mechanisms.

Recent studies have confirmed that the angiogenic potential of ADSCs is associated with VEGF [32, 47]. Using an ADSCs/endothelial cell co-culture model, Xie et al. [32] found that VEGF may be responsible for the enhancement of angiogenesis triggered by low O_2_ tension. Our results showed that, the expression level of VEGF was significantly enhanced on day 7 in the hypoxia group. We also found no significant difference in VEGF expression between the normoxia and hypoxia groups on day 3. We suggest that, under hypoxia, the ADSCs underwent a prolonged lag period for adapting to different culture environments, although the exact mechanisms remain to be clarified.

Nevertheless, the present study has several limitations. First, the effects of hypoxic conditions on tenogenic differentiation were not quantitated at protein levels. Second, the influences of longer exposure periods (> 7 d) on ADSCs proliferation, migration, and tenogenic differentiation were not determined. These will be investigated in further studies.

## Conclusions

In conclusion, our findings demonstrated that long-term exposure to 5% O_2_ reduced the proliferation and migration (vs. 2% O_2_) of rat ADSCs, and significantly downregulated HIF-1α expression. Additionally, 5% O_2_ significantly enhanced the tenogenic differentiation of ADSCs via an HIF-1α signaling pathway, and significantly activated their VEGF expression. Given these advantages of hypoxia, it may be a promising condition for tendon tissue engineering based on ADSCs.
